# Orexin-A and motion sickness: a systematic review of animal model studies

**DOI:** 10.3389/fphar.2025.1624080

**Published:** 2025-06-30

**Authors:** Xu Cai, Long Zhao, Xin Wang, Jiahui Chen, Ying Yuan, Biao Gao, Yanli You

**Affiliations:** ^1^ School of Traditional Chinese Medicine, Naval Medical University, Shanghai, China; ^2^ The 988th Hospital of PLA Joint Logistics Support, Zhengzhou, Henan, China; ^3^ Teaching and Research Support Center, Naval Medical University, Shanghai, China

**Keywords:** orexin-A, motion sickness, systematic review, neural mechanisms, therapeutic potential

## Abstract

**Background:**

Sensory input mismatches among the vestibular system, autonomic control, and visual perception cause motion sickness. Anticholinergics and antihistamines are commonly used but have limited efficacy and cause significant side effects. Orexin-A, a hypothalamic neuropeptide, has recently garnered attention for its potential role in controlling motion sickness.

**Objective:**

To summarize current knowledge on the effects and mechanisms of orexin-A in reducing motion sickness, identify gaps, and propose future research directions.

**Methods:**

Five qualified animal experiments were identified after searching PubMed, Scopus, Cochrane Library, Embase, and WoS. The SYRCLE tool was used to evaluate study quality, followed by a qualitative synthesis.

**Results:**

Orexin-A reduced motion-induced behavioral abnormalities, nausea, and vomiting in rat and cat models. These benefits are likely mediated by the modulation of hypothalamic nuclei activity, enhanced stomach motility, and improved vestibular function. However, several limitations were observed, including inadequate reporting on randomization, blinding, and allocation concealment, as well as heterogeneity in interventions and outcome measures.

**Conclusion:**

Animal model studies indicates that orexin-A mitigates motion sickness (MS) symptoms in animal models, but overall certainty is low to moderate owing to risk of bias and indirectness. Rigorous, blinded studies with standardized outcomes—and ultimately, early-phase clinical trials, are needed to clarify therapeutic potential.

## Highlights

• Initial evidence supporting orexin-A’s role in reducing motion sickness symptoms and its underlying mechanisms comes from a systematic review of animal studies.
• Orexin-A effectively modulates hypothalamic nuclei (ARC, PVN), vagal outflow, and vestibular function, with distinct regulatory roles for the OX1R/OX2R receptor subtypes, thereby reducing nausea, vomiting, and motor disturbances.
• Nasal delivery and various intervention techniques highlight orexin-A’s potential as a novel anti-motion sickness agent due to its quick and sensible approach.
• Data obtained from animal models emphasize the need for clinical studies and further mechanistic research to confirm orexin-A’s efficacy and safety in humans.

## 1 Introduction

Motion sickness is caused by the failure of the central nervous system to accurately integrate and regulate discordant sensory inputs from the vestibular system, autonomic pathways, and visual perception. Clinically, it is characterized by nausea, vomiting, dizziness, and sweating (Golding, 2006; Zhang et al., 2016). Studies have shown differences in environment and individual lead to variability in onset of motion sickness (Sharma and Aparna, 1997), with the highest incidence (50.2%) observed in young individuals (Strupp et al., 2018). Beyond affecting daily lives and work efficiency, motion sickness significantly impairs the performance of drivers (Pawar et al., 2023), astronauts (Heer and Paloski, 2006), and military personnel (Powell-Dunford and Bushby, 2017).

Current intervention primarily targets peripheral or single neurotransmitter systems, such as anticholinergics and antihistamines. They mostly have limited efficacy accompanying marked side effects (Karrim et al., 2022). Therefore, there is an urgent need to deepen our understanding of central regulatory circuits and molecular mechanisms to develop safer and more efficient therapeutic strategies.

Recently, the physiological roles of the hypothalamic neuropeptide orexin-A (hypocretin-1) have garnered considerable interest. Secreted by hypothalamic neurons, orexin-A acts through OX1R and OX2R (Soya and Sakurai, 2020), mediating a broad range of central regulatory effects, including modulation of arousal, energy metabolism, emotional states, and stress responses (Sakurai et al., 1998; Sakurai, 2002; Samson et al., 2005). Evidence suggests orexin projections to vestibular nuclei, the solitary tract nucleus, and other autonomic control centers play roles in regulating the central mechanisms underlying motion sickness (Okumura and Nozu, 2011; Marcus et al., 2005; Ben Musa et al., 2024; Pan et al., 2016). However, a systematic review of orexin-A’s specific regulatory pathways and action profiles in motion sickness is still lacking. Although animal models hint at the orexin’s involvement in nausea, vomiting, and abnormal motor behaviors associated with motion sickness (Okumura and Nozu, 2011), these findings remain scattered and lack clinical support, leaving precise mechanisms and translational potential of orexin-A still not fully understood.

While our initial inclusion criteria allowed various preclinical (including animal and cell-based) and clinical research, the systematic search yielded only *in vivo* animal model studies; no relevant *in vitro* or human data were identified. Consequently, this review focuses exclusively on animal experiments to synthesize current evidence and generate hypotheses for future translational research.

Additionally, individuals with vestibular lesions exhibit reduced susceptibility to motion sickness (Strupp et al., 2018). Orexin-A has demonstrated potential benefits in two pathological contexts—vestibular dysfunction and chemotherapy-induced nausea and vomiting (CINV). Specifically, vestibular damage alters orexin-A expression and induces abnormal motor behavior in rats, which can be ameliorated by exogenous orexin-A (Pan et al., 2016). In cisplatin-induced anorexic rats, orexin neuropeptide expression in the hypothalamus is upregulated (Yoshimura et al., 2013), and studies indicate that orexin-A relieves nausea and vomiting symptoms via regulation of the hypothalamic arcuate nucleus–paraventricular nucleus (ARC-PVN) pathway (Guo et al., 2018; Guo et al., 2019). These findings suggest that the orexin system may serve as an important neural circuit connecting vestibular function and the emetic reflex.

Despite insights (Pan et al., 2024), several issues remain unresolved. First, current research focuses on animal models, and has yet to form consistent conclusions, with clinical translatability needing further validation. Second, the precise molecular mechanisms through which orexin-A modulates motion sickness, including its interactions with other neurotransmitter systems, have not yet been fully elucidated (Singh and Biswas, 2023).

Therefore, systematic and comprehensive evaluation of studies on orexin-A in motion sickness, with a particular focus on central mechanisms in vestibular and hypothalamic nuclei and role in regulating gastrointestinal function, is essential, identifying gaps and informing future directions. This review aims to integrate studies’ result, analyze the central circuit mechanisms by which orexin-A alleviates motion sickness, explore the differential regulatory functions of OX1R and OX2R receptor subtypes, and highlight evidence gaps and unresolved questions. Ultimately, this framework will help lay the theoretical foundation for running high-quality clinical trials and developing effective interventions for motion sickness.

## 2 Methods

### 2.1 Database

A comprehensive and systematic literature search was conducted to identify studies investigating the relationship between orexin-A and motion sickness from the inception of databases up to February 2025. To ensure extensive coverage of relevant biomedical and clinical research, five major electronic databases were selected: PubMed, Scopus, Cochrane Library, Embase, and Web of Science. These databases were chosen for their wide-ranging repositories and authoritative content in the fields of neuroscience, pharmacology, and clinical medicine.

### 2.2 Search strategy

The search strategy was meticulously developed using a combination of Medical Subject Headings (MeSH) and relevant keywords related to orexin-A and motion sickness. Boolean operators (AND, OR) were employed to refine the search and enhance the retrieval of pertinent studies. The primary keywords and their combinations included: Orexin-A OR hypocretin-1; motion sickness OR kinetosis.

These terms were interconnected using the AND operator to ensure the inclusion of studies addressing both orexin-A and motion sickness. Detailed search strategies, including specific Boolean logic and keyword permutations, are provided in Appendix A.

### 2.3 Selection and screening

The initial search across all databases yielded a total of 599 records (PubMed: 15, Scopus: 224, Cochrane Library: 33, Embase: 220, Web of Science: 107). These records were imported into the reference management software NoteExpress (V4.1.0.9990) to facilitate organization and screening. Duplicate records identified across multiple databases were removed, resulting in 342 unique records.

#### 2.3.1 Initial screening

Two independent reviewers conducted an initial screening based on the titles and abstracts of the 342 unique records. Studies were excluded if they did not pertain to orexin-A or motion sickness, were not original research or relevant reviews, or were published in a language other than English. Any discrepancies between reviewers were resolved through discussion or by consulting a third reviewer.

#### 2.3.2 Full-text assessment

Following the initial screening, the full texts of the remaining 50 potentially relevant studies were retrieved and assessed against the inclusion and exclusion criteria. This thorough evaluation included 5 studies (Pan et al., 2016; Guo et al., 2018; Guo et al., 2019; Pan et al., 2024; Yuan et al., 2021) in the systematic review.

### 2.4 Inclusion and exclusion criteria

#### 2.4.1 Inclusion criteria

• Study Types: Original research articles (including animal studies, cell studies, and/or clinical research), reviews, and meta-analyses.
• Topic Relevance: Studies investigating the relationship between orexin-A and motion sickness, encompassing mechanisms, therapeutic effects, or physiological impacts.
• Language: Publications written in English.
• Publication Status: Full-text articles published in peer-reviewed journals.

#### 2.4.2 Exclusion criteria

• Inappropriate Study Designs: Conference abstracts, letters, patents, editorials, or protocols without reported results.
• Duplicates: only the most recent and comprehensive version was included.
• Quality Issues: Studies with serious methodological flaws or unreliable data.

Note on Study Type Inclusion: Although we originally intended to include both preclinical (animal and cell) and clinical research, our final selection yielded only animal studies that met all inclusion criteria. The absence of clinical studies suggests a research gap in translating preclinical findings into human investigations.

### 2.5 Additional search methods

To enhance the comprehensiveness of the review, the reference lists of included studies and relevant reviews were manually searched for additional pertinent articles. Expert consultations were also conducted to identify unpublished studies or unpublished studies. No additional studies meeting the inclusion criteria were identified by these methods.

### 2.6 Quality assessment

Studies were assessed using SYRCLE’s Risk of Bias tool (Hooijmans et al., 2014), which adapts the Cochrane risk of bias assessment methodology for animal research. This tool evaluates ten domains, such as random sequence generation, baseline balance, allocation concealment, blinding and outcome assessors, completeness of data, selective reporting, and other potential sources of bias. Two reviewers independently evaluated each study by extracting all available information on these domains. When primary studies did not report key methodological details (e.g., randomization procedure, blinding, allocation concealment), the domain was recorded as “unclear,” and this was subsequently highlighted as a limitation in the discussion. Any discrepancies in the quality assessments were resolved through discussion or by consulting the third. A brief description of each domain and the criteria used to judge the risk of bias is provided in Appendix B. This systematic quality assessment ensures transparency and allows readers to understand how missing methodological information may affect reproducibility and confidence in the findings. Each SYRCLE domain was converted to a numeric score - Low = 0, Moderate = 0.5, Unclear = 1, and High = 2 - following a pre-specified domain-count rule. For every study, the sum of domain scores was divided by the maximum possible score (2 × number of applicable domains) and expressed as a percentage. Overall risk of bias was then categorized as Low (≤25%), Moderate (>25%–50%), Unclear (>50%–75%), or High (>75%).

### 2.7 Meta analysis

Although most endpoints could not be combined due to differences in study design, two outcomes—kaolin intake and gastric motility index (%MI)—were identified as suitable for quantitative synthesis. We performed meta-analyses on these endpoints using the R meta package, extracting group sample sizes, means, and standard deviations to calculate standardized mean differences (SMD) under both fixed-effect and random-effects models, and we assessed heterogeneity (I^2^ and Q-test).

### 2.8 Adherence to reporting guidelines

**FIGURE 1:**
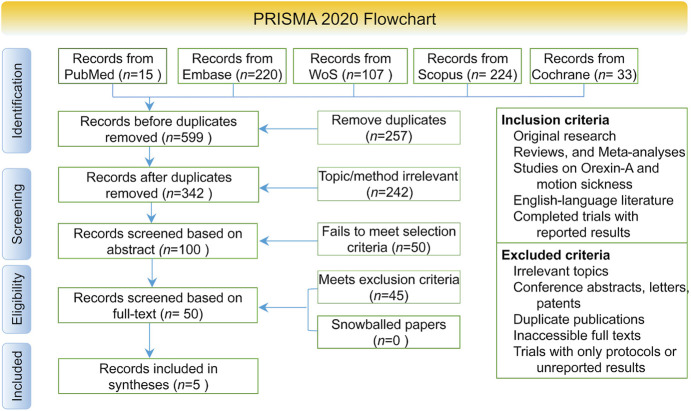
Flowchart of research literature acquisition.

This systematic review was conducted in accordance with the Preferred Reporting Items for Systematic Reviews and Meta-Analyses (PRISMA) 2020 guidelines (Page et al., 2021). The PRISMA flow diagram detailing the study selection process is presented in Figure 1.

### 2.9 Data extraction

For each included study, the following data were extracted using a standardized form: Authors and publication year, study design and type, sample size and characteristics, orexin-A dose or treatment protocol, motion sickness assessment methods, key findings, and outcomes. This information was systematically compiled to facilitate comprehensive analysis and synthesis of the results between studies.

### 2.10 Ethical considerations

Ethical approval was not required as this study is a systematic review and does not involve collecting new data from human or animal subjects.

## 3 Result

### 3.1 Literature inclusion

The initial database search identified 599 records, of which 342 remained after removing duplicates. Following title and abstract screening and a thorough full-text evaluation, five studies met the inclusion criteria and were ultimately incorporated into this systematic review (Figure 1). The screening process was conducted in accordance with the PRISMA guidelines.

### 3.2 Basic characteristics of included studies

All five included studies were conducted using animal models, primarily rats (with one study also involving cats), to investigate the role of orexin-A in motion sickness and associated symptoms such as nausea, vomiting, gastric motility disturbances, and abnormal motor behaviors. Published between 2016 and 2025 (Table 1), these studies employed various interventions, including central (intracerebroventricular or intracranial nucleus) and peripheral administration routes, as well as comparative agents such as SB-334867, TCS-OX2-29, cisplatin, and seabuckthorn seed oil. The measured outcomes encompassed behavioral parameters (e.g., vomiting frequency, feeding behavior, locomotor activity), physiological indicators (e.g., gastric emptying rate, body temperature), and neurobiological markers (e.g., neuronal firing rates, Fos protein expression, and orexin-related gene and protein levels).

**TABLE 1:** Basic characteristics of included studies.

No.	Author (Year)	Research objective	Experimental model	Experimental design	Intervention	Control measures	Main outcome indicators
1	(Guo et al., 2018)	To explore the effect of orexin-A from the arcuate nucleus on cisplatin-induced nausea and vomiting in rats, and the changes in hypothalamic paraventricular nucleus neuron activity	Male Wistar rats, *n* = 10/group, total 60 rats	RCT, blinding not specified; Experiment: Intraperitoneal injection of cisplatin (6 mg/kg, single dose)	Drugs: Orexin-A, SB-334867; Dosage: Arcuate nucleus injection of orexin-A 0.5 μg, SB-334867 5.0 μg	NS	Kaolin consumption, gastric motility, neuronal activity (neuron firing rate), etc.
2	(Guo et al., 2019)	To study the effect of orexin-A from the arcuate nucleus on cisplatin-induced gastric motility disorders	Male Wistar rats, *n* = 10/group, total 50 rats	RCT, blinding not specified; Experiment: Intraperitoneal injection of cisplatin (6 mg/kg, single dose)	Drugs: Orexin-A, SB-408124, BIBO3304; Dosage: Arcuate nucleus injection of orexin-A 0.5 μg, SB-408124 5.0 μg, third ventricle injection of BIBO3304 60 μg	NS	Gastric emptying rate, gastric motility indicators, vagus nerve firing frequency
3	(Pan et al., 2024)	To investigate the mechanism of orexin-A in motion sickness	Adult male Sprague–Dawley rats, *n* = 8/group (behavioral experiments), *n* = 5/group (Fos expression), total of 1,024 rats; Adult male domestic shorthair cats, *n* = 24 (repeated-measures design)	RCT, blinding not specified; Experiment: Rotational stimulation protocol	Drugs: Orexin-A, SB-334867, TCS-OX2-29; Dosage: Intracerebroventricular (Orexin-A: 20, 10 μg; SB-334867: 15, 10, and 5 μg), brain nuclei (Orexin-A: 4 μg, SB-334867: 6 μg, TCS-OX2-29: 5 μg), nasal administration (cats, orexin-A: 60 and 120 μg/kg, scopolamine: 20 μg/kg), single dose	NS, rotational group vs. stationary group, baseline reference, positive drug control, gene expression control	Behavioral indicators: Feeding behavior, spontaneous movement, conditioned mouth opening reflex; Physiological indicators: Temperature changes; Molecular biology indicators: Fos protein expression; Specific indicators for cat experiments: Vomiting response, salivation response, motion sickness behavior score; Neuronal activity indicators: Neuronal activity in specific brain nuclei, changes in neuron firing rate
4	(Pan et al., 2016)	To study changes in the orexin/hypocritic system after vestibular injury and its effects on motor dysfunction	Male Sprague–Dawley rats, *n* = 6/group (OXA), *n* = 8/group (JNJ7777120), *n* = 8/group (SB334867), total 200 rats	RCT, blinding not specified; Experiment: Experimental vestibular injury model	Intraperitoneal injection: JNJ7777120 (20 mg/kg); Intracerebroventricular injection: SB334867 (16 µg)	Vehicle solution	Motor behavioral indicators, orexin expression levels
5	(Yuan et al., 2021)	To evaluate the preventive effects of sea buckthorn seed oil on cisplatin-induced nausea and vomiting and its impact on orexin-A	Male Wistar rats, *n* = 18/group (behavioral experiments), *n* = 8/group (gastric motility assessment), *n* = 8/group (molecular biology tests), *n* = 2/group (immunohistochemical analysis), total of 108 rats	RCT, blinding not specified; Experiment: Intraperitoneal injection of cisplatin (6 mg/kg, single dose)	Sea buckthorn seed oil gavage, Dosage: 0.850, 1.675, 3.350 g/kg, for 6 days; Ondansetron gavage, Dosage: 2 mg/kg, same administration method as SSO, continued for 6 days	Negative control: NS (NCG and CG groups); Positive control: Ondansetron (2 mg/kg) (OG group)	Vomiting frequency, gastric emptying rate, plasma orexin-A levels, mRNA and protein expression of OX1R in hypothalamus and brainstem, OXA expression in the lateral hypothalamic area (LHA), HPLC analysis of SSO components

Note: RCT: Randomized controlled trial. NS: saline (0.9%w/v).

### 3.3 Quality assessment

A quality assessment of the five included animal studies was conducted using the SYRCLE’s Risk of Bias tool (Table 2). Overall, the studies exhibited certain reporting deficiencies, particularly regarding details of randomization, allocation concealment, and blinding procedures, leading to unclear or moderate risks of bias in these domains. Nevertheless, all studies performed well regarding data completeness and outcome reporting, with no apparent evidence of missing data or selective reporting bias.

**TABLE 2:** Risk of bias assessment for included studies.

No.	Authors (Year)	Random sequence generation	Baseline characteristic balance	Allocation concealment	Blinding of researchers	Blinding of outcome assessors	Random outcome assessment	Attrition bias	Reporting bias	Animal housing	Ethical approval	Overall risk
1	(Guo et al., 2018)	Moderate	Low	Unclear	Unclear	Low	Low	Low	Low	Low	Low	Low
2	(Guo et al., 2019)	Moderate	Unclear	Unclear	Unclear	Unclear	Unclear	Low	Low	Low	Low	Moderate
3	(Pan et al., 2024)	Moderate	Low	Unclear	Low	Low	Low	Low	Low	Low	Low	Low
4	(Pan et al., 2016)	Moderate	Unclear	Unclear	Unclear	Low	Low	Low	Low	Low	Low	Low
5	(Yuan et al., 2021)	Moderate	Unclear	Unclear	Unclear	Unclear	Unclear	Low	Low	Low	Low	Moderate

Assessment tool: SYRCLE, risk-of-bias scale for animal studies, covering 10 domains—sequence generation, baseline characteristic balance, allocation concealment, random housing, blinding of caregivers/investigators, random outcome assessment, blinding of outcome assessors, attrition bias, selective outcome reporting, and other sources of bias. Judgement categories: Low risk, Moderate risk, Unclear, High risk. Domain-count scoring rule: Low = 0, Moderate = 0.5, Unclear = 1, High = 2; domains marked Not applicable are excluded from both numerator and denominator. Overall risk thresholds: ≤ 25% → Low; >25–50% → Moderate; >50–75% → Unclear; >75% → High.

Assessment Explanation: (1) Random sequence generation: Although all included studies mentioned ‘random grouping’, none provided explicit randomization procedures, resulting in a judgment of moderate risk. (2) Baseline characteristic balance: The studies did not report detailed baseline characteristics (e.g., body weight, age) for each group, leading to an unclear risk judgment. (3) Allocation concealment: None of the studies mentioned any allocation concealment measures, thus deemed unclear. (4) Blinding of researchers: If studies did not state whether researchers were blinded to group assignment during the experiment, the risk was assessed as unclear. (5) Blinding of outcome assessors: The risk was deemed unclear if studies did not indicate whether outcome assessors were blinded to group assignment. (6) Completeness of the outcome data: All studies fully reported outcome data without sample attrition or missing data, resulting in a low-risk assessment. (7) Selective reporting: All studies reported their predefined primary outcomes, and no evidence of selective reporting was identified. Therefore, reporting bias was judged to be low risk. (8) Animal welfare and ethics: All studies claimed adherence to animal welfare and ethical standards, resulting in a low-risk judgment.

Overall Assessment: The five included studies showed insufficient reporting in key areas such as randomization procedures, blinding, and allocation concealment, raising concerns about selection, performance, and detection biases. These factors may compromise the reliability and internal validity of the findings. However, all studies fully reported their outcome data without apparent selective reporting or data loss. Their compliance with animal housing standards and ethical approvals further supports the credibility of the research.

Recommendations: (1) Improved study design: Implement rigorous randomization methods, clearly describe the randomization process, and detail allocation concealment procedures. (2) Blinding: Ensure that researchers and outcome assessors are blinded to group assignments to minimize subjective bias whenever feasible. (3) Comprehensive reporting: Adherence to the ARRIVE guidelines (Kilkenny et al., 2010) for transparent reporting of experimental design, procedures, and results, thus improving overall study quality and credibility of the study.

### 3.4 Main results

#### 3.4.1 Guo F et al. (2018)

Objective: Investigate the effects of ARC (arcuate nucleus) on cisplatin-induced nausea and vomiting in rats and changes in PVN neuronal activity (paraventricular nucleus) neuronal activity (Guo et al., 2018).

Key Results: (1) Vomiting frequency: Significantly reduced in the orexin-A group compared to controls (*P* < 0.01). (2) Nausea behavior scores: Significantly lower in the orexin-A group (*P* < 0.01). (3) PVN neuronal firing rate: Significantly increased in orexin-A-treated rats (*P* < 0.05). (4) Expression of the c-Fos protein: Increased c-Fos-positive neurons in the PVN, indicating enhanced neuronal activation (*P* < 0.05).

Conclusions: ARC injection of orexin-A alleviates cisplatin-induced nausea and vomiting in rats, likely by activating hypothalamic PVN neurons. These findings suggest that orexin-A may mitigate chemotherapy-related nausea and vomiting by modulating hypothalamic neuronal activity.

#### 3.4.2 Guo F et al. (2019)

Objective: To examine the effects of ARC injection of orexin-A on cisplatin-induced gastric motility disturbances in rats (Guo et al., 2019).

Key Results: (1) Gastric emptying rate: Significantly higher in the orexin-A treatment group compared to controls (*P* < 0.01). (2) Gastric antrum contraction amplitude and frequency: Markedly increased following orexin-A administration. (3) Vagal nerve activity: In cisplatin-treated rats, gastric distension-inhibitory (GD-I) vagal neuronal firing frequency increased after ARC administration of orexin-A (*P* < 0.05).

Conclusions: ARC injection of orexin-A improves cisplatin-induced gastric motility disorders and promotes gastric emptying, potentially by activating vagal efferent pathways.

#### 3.4.3 Pan L et al. (2024)

Objective: Explore the effects of central orexin-A administration on motion sickness-related behaviors and hypothalamic neuronal activity in rats (Pan et al., 2024).

Key Results: (1) Intracerebroventricular (ICV) administration: (i) Behavioral effects: Dose-dependent improvement in appetite, enhanced locomotor activity (increased total travel distance, activity time, and frequency), suppression of conditioned gaping responses, and attenuation of hypothermia. (ii) Neuronal activity changes: Decreased Fos expression in DMV and PVN, increased Fos expression in PMV. (2) Receptor antagonist experiments: (i) OX1R antagonist (SB-334867): Blocked the orexin-A-induced improvements in appetite and activity, as well as the suppression of conditioned gaping. (ii) OX2R antagonist (TCS-OX2-29): Blocked the orexin-A-mediated thermoregulatory effects. (3) Nucleus-specific injections: (i) DMV injection: Increased food intake and activity. (ii) PVN injection: Inhibited conditioned gaping responses. (iii) PMV injection: Involved in temperature regulation. (4) Nasal administration in cats: Orexin-A exhibited antiemetic effects comparable to scopolamine, superior appetite improvement, and fewer side effects such as drooling. (5) Changes in neuronal firing rates: (i) DMV neurons: Rotation significantly increased their firing rates; Orexin-A suppressed this overexcitation, an effect negated by OX1R antagonists. (ii) PVN neurons: Rotation elevated PVN neuronal firing; Orexin-A reduced this excessive activity, and OX1R antagonist pretreatment abolished orexin-A’s inhibitory effect. (iii) PMV neurons: Baseline firing decreased post-rotation; Orexin-A enhanced PMV neuronal firing, an effect blocked by OX2R antagonists.

Conclusions: Orexin-A improves motion sickness through coordinated modulation of multiple brain regions and different receptor subtype functions. Nasal administration emerges as a potential therapeutic strategy with fewer side effects than scopolamine. Behavioral improvements, changes in neuronal firing, and altered Fos expression collectively confirm orexin-A’s multilevel regulatory effects.

#### 3.4.4 Pan L et al. (2016)

Objective: Investigate changes in the orexin/Hypocretin system following vestibular lesions and its role in abnormal motor behaviors (Pan et al., 2016).

Key Results: (1) Motor behavior abnormalities: Vestibular lesion-induced rats showed impaired balance and unstable gait (*P* < 0.01). (2) Hypothalamic orexin-A expression: Significantly elevated in vestibular lesion rats (*P* < 0.05). (3) Orexin receptors: OX1R antagonists alleviated behavioral symptoms induced by vestibular lesions.

Conclusions: Vestibular lesions may alter orexin-A expression and are closely associated with hyperactivity and altered exploratory behaviors. Pharmacological intervention with H4R antagonists (JNJ7777120) and OX1R antagonists (SB334867) significantly reduced behavioral abnormalities induced by vestibular lesions, suggesting that the orexin system plays a crucial role in vestibular lesion-related motor and behavioral changes.

#### 3.4.5 Yuan W et al. (2021)

Objective: To evaluate the preventive effects of seabuckthorn seed oil (SSO) on cisplatin-induced nausea and vomiting and to investigate its influence on orexin-A levels (Yuan et al., 2021).

Key Results: (1) Vomiting frequency: Rats treated with SSO exhibited a significantly lower vomiting frequency compared to controls (*P* < 0.001). (2) Plasma orexin-A levels: Plasma orexin-A concentrations were significantly elevated in the SSO group (*P* < 0.01). (3) Gastric emptying rate: SSO significantly increased gastric emptying rates (*P* < 0.01). (3) Antioxidant indicators: Rats receiving SSO showed enhanced antioxidant enzyme activities and reduced oxidative stress levels (*P* < 0.05).

Conclusions: SSO alleviates cisplatin-induced vomiting and delayed gastric emptying, potentially by elevating orexin-A levels and OX1R expression, thereby exerting protective effects through the orexin system.

### 3.5 Synthesis of findings

**FIGURE 2:**
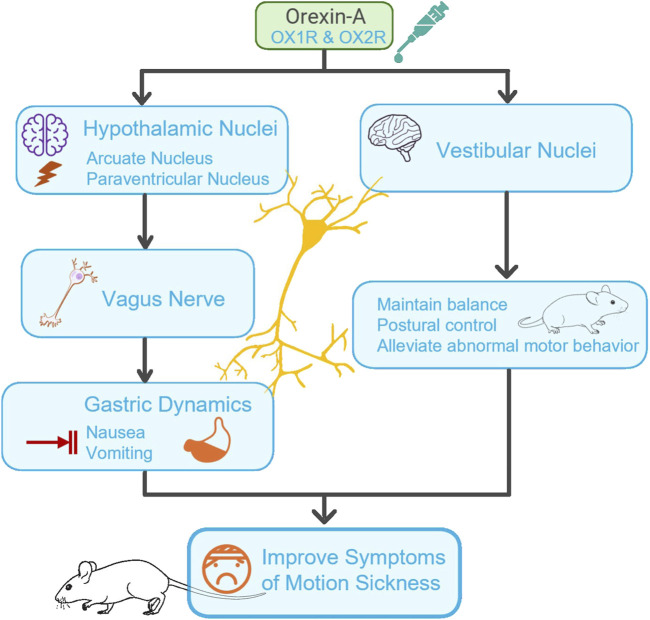
Mechanism of Orexin-A in regulating motion sickness via central-peripheral pathways. This figure illustrates the regulation of central neural circuits by orexin-A in hypothalamic nuclei (such as the arcate nucleus (ARC) and paraventricular nucleus (PVN)). It shows how orexin-A affects gastric motility through the Vagus nerve and regulates the vestibular nucleus to maintain balance and postural stability, thereby improving overall symptoms of motion sickness (such as nausea, vomiting, and abnormal motor behavior). Arrows indicate the direction of action and signal transmission pathways.

Despite variations in experimental approaches and models, the included studies consistently support orexin-A’s potential role in alleviating motion sickness and its associated symptoms. The underlying mechanisms can be conceptualized as a coordinated, multilevel interplay among various pathways (Figure 2). On the one hand, orexin-A targets specific hypothalamic nuclei (e.g., ARC, PVN), altering neuronal firing patterns and modulating gastric motility via vagal efferent pathways, thus improving gastric emptying and mitigating nausea and vomiting. Simultaneously, orexin-A modulates activity in vestibular nuclei (e.g., the medial vestibular nucleus) and related integrative centers such as the dorsal motor nucleus of the vagus, the nucleus of the solitary tract, and hypothalamic regions involved in vestibular processing. This modulation may contribute to maintain balance and postural stability, thereby reducing abnormal motor behaviors observed in motion sickness models. This multi-tier central-peripheral network of actions provides foundational evidence that orexin-A may serve as a promising therapeutic target for managing motion sickness.

### 3.6 Heterogeneity

The included studies exhibit notable heterogeneity in animal models, inducing stimuli (e.g., cisplatin administration, rotational stimulation, vestibular lesions), intervention methods (central versus peripheral administration, varying doses and durations), and outcome measures. These factors may limit the comparability of results. Furthermore, no clinical evidence currently supports the findings, underscoring the need for future human studies to confirm orexin-A’s therapeutic potential in clinical settings.

### 3.7 Meta analysis

**FIGURE 3:**
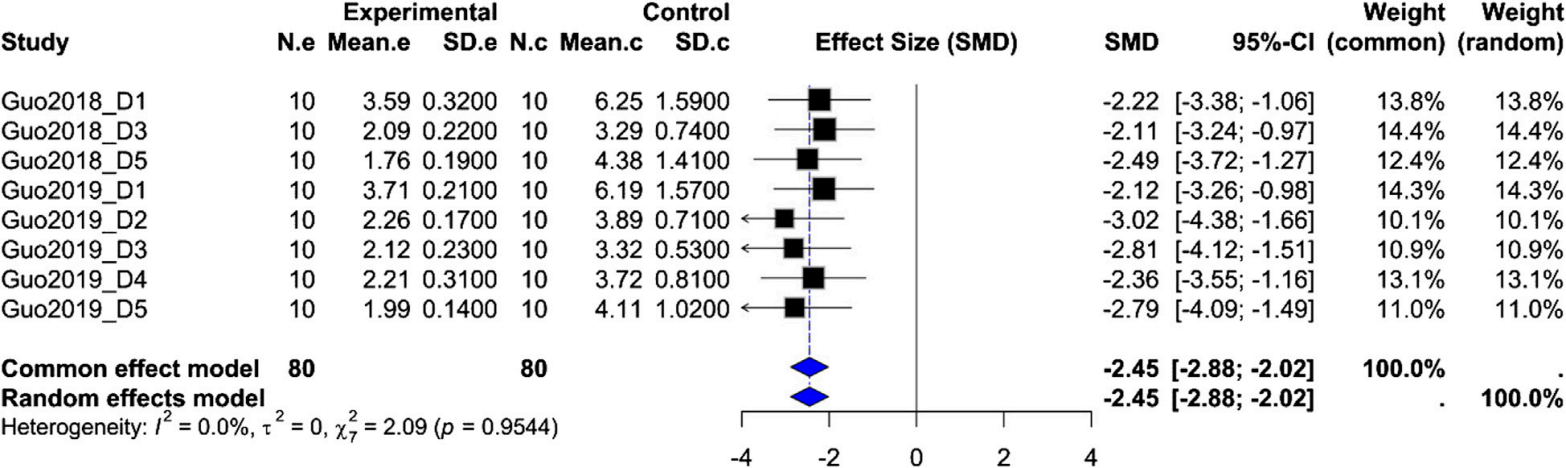
Meta-analysis of Orexin-A effect on kaolin intake in wistar rats. Forest plot of the effect of Orexin-A on Kaolin intake in Wistar rats compared to controls, based on a random-effects model. The pooled standardized mean difference (SMD) using Hedges’ g was −2.45 (*P* < 0.0001). Heterogeneity: τ^2^ = 0.0, *I* ^2^ = 0.0% (*P*_Q = 0.95).

Kaolin intake: Based on eight groups from two studies (orexin-A *n* = 80 vs. control *n* = 80), the pooled SMD = −2.45 (95% CI –2.88, –2.02; Z = −11.13; *P* < 0.0001) with negligible heterogeneity (*I* ^2^ = 0%). Both fixed-effect and random-effects models yielded consistent results, indicating that ARC injection of orexin-A significantly reduces cisplatin-induced pica behavior (Figure 3).

**FIGURE 4:**
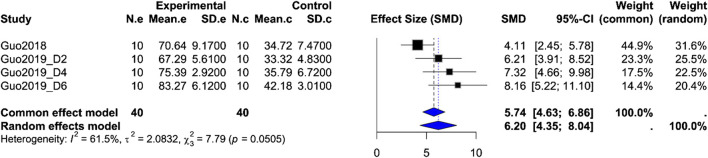
Meta-analysis of Orexin-A effect on gastric motility index in wistar rats. Forest plot of the effect of Orexin-A on gastric motility index (%MI) in Wistar rats compared to controls, based on a random-effects model. The pooled standardized mean difference (SMD) using Hedges’ g was 6.20 (*P* < 0.0001). Heterogeneity: τ^2^ = 2.08; *I* ^2^ = 61.5%, *P*_Q = 0.0505.

Gastric motility index (%MI): Based on four rat groups from the same two studies (orexin-A n = 40 vs. control *n* = 40), the pooled SMD = 6.20 (95% CI 4.35, 8.04; Z = 6.59; *P* < 0.0001), but with moderate-to-high heterogeneity (*I* ^2^ = 61.5%, *P*_Q = 0.0505). Both fixed-effect and random-effects models show a significant enhancement of gastric motility after ARC injection of orexin-A, although variability in study design may influence effect size (Figure 4).

## 4 Discussion

### 4.1 Key findings

Combining data from five animal studies, this systematic review gave initial validation of orexin-A’s possible ability to reduce motion sickness and related symptoms (e.g., nausea, vomiting, and aberrant motion behavior). Although the included studies used different models and intervention, their results imply that orexin-A reduces motion sickness symptoms by central (hypothalamic nuclei) and peripheral (vagal-gastrointestinal) pathways. These initial findings provide a theoretical basis for considering orexin-A as a novel approach in managing motion sickness.

The meta-analyses of kaolin intake and gastric motility index both show highly significant effects under fixed-effect and random-effects models, providing quantitative support that ARC injection of orexin-A substantially reduces cisplatin-induced pica behavior and enhances gastric motility. However, the extremely low heterogeneity for kaolin intake suggests consistency in measurement and methods across studies, whereas moderate-to-high heterogeneity for %MI likely reflects differences in dose, measurement time points, experimental protocols, or animal handling. Therefore, these results must be interpreted with caution. Although these analyses offer preliminary quantitative evidence for orexin-A’s effects, they are based on only two studies and limited endpoints, precluding broadly generalizable conclusions. As more uniformly designed preclinical studies become available, larger-scale meta-analyses across additional endpoints will be possible.

### 4.2 Underlying mechanism

Several studies have focused on the mechanisms by which orexin-A acts on specific hypothalamic nuclei. Orexinergic neurons project extensively to the arcuate nucleus (ARC), paraventricular nucleus (PVN), and other central structures, influencing neuronal firing patterns and the release of neurotransmitters (Peyron et al., 1998). Guo F et al. (Guo et al., 2018; Guo et al., 2019) demonstrated that orexin-A enhances PVN neuronal activity, which is shown by much higher firing rates and upregulated c-Fos expression—so lowering sensitivity to nausea and vomiting. Meanwhile, Pan L et al. (Pan et al., 2024) reported orexin-A simultaneously modulates multiple nuclei, including DMV, PMV, and PVN. This coordinated regulation affects multiple levels of behavior, body temperature, and neural activity.

Peripherally, Orexin-A’s modulation of gastric motility is particularly notable. It alleviates cisplatin-induced gastric dysmotility, enhances gastric emptying, and reduces vomiting frequency. These findings are consistent with the known orexinergic facilitation of feeding behavior (Sakurai et al., 1998; Yoshimura et al., 2013) and indirectly influence central and peripheral pathways through the vagal efferent circuits.

Furthermore, Orexin-A’s impact on vestibular function provides new insights into the pathogenesis of motion sickness. Pan L et al. (Pan et al., 2016) showed that vestibular lesions reduced orexin-A and receptor expression levels, leading to abnormal motor behaviors. By adjusting vestibular system activity, orexin-A mitigates balance disturbances and gait instability triggered by vestibular damage, thereby maintaining normal motor function.

### 4.3 Consistency with existing literature

The findings of this review are consistent with previous understandings of the orexin system’s multifaceted regulatory roles (Soya and Sakurai, 2020; Sakurai et al., 1998; Sakurai, 2002; Samson et al., 2005; Okumura and Nozu, 2011). Despite variations in experimental models, doses, and administration routes - ranging from ARC injections to intracerebroventricular and nasal delivery (Guo et al., 2018; Guo et al., 2019; Pan et al., 2024), the general trend supports the ability of orexin-A to integrate modulation in both central and peripheral pathways. Including seabuckthorn seed oil interventions (Yuan et al., 2021) extends the range of potential strategies and provides other pathways for next clinical applications.

To place orexin-A/ARC–PVN signaling in the context of established antiemetic mechanisms, we considered its potential interactions with serotonergic (5-HT_3_) and neurokinin-1 (NK_1_) pathways. Although direct preclinical data are scarce, orexinergic modulation of autonomic and gastrointestinal circuits may overlap with 5-HT_3_–mediated and NK_1_–mediated processes underlying nausea and vomiting (Guo et al., 2018; Kirchgessner, 2002; Mavanji et al., 2022; Messina et al., 2014). Future preclinical studies should therefore evaluate orexin-A in combination with 5-HT_3_ or NK_1_ receptor antagonists, to assess additive or synergistic effects on emesis proxies and physiological endpoints. In addition, potential off-target central effects of orexin-A—such as increased arousal or metabolic changes—should be systematically monitored to inform safety profiles.

### 4.4 Clinical opportunities

The marked effectiveness of orexin-A in reducing motion sickness symptoms in animal models creates promising opportunities for translating these findings into human studies. Compared to conventional anticholinergic and antihistaminic medications, orexin-A may reduce common side effects such as drowsiness and fatigue. Its suitability for nasal administration, characterized by rapid onset, noninvasiveness, and high patient acceptability—holds particular promise for individuals requiring immediate symptom relief, including astronauts, military personnel, and professional drivers. Moreover, combining orexin-A with current treatments like ondansetron warrants further research to improve therapeutic efficacy while reducing side effects.

We recommend first assessing the safety and pharmacokinetic profile of orexin-A administration; conducting early-phase clinical trials in individuals at high risk for motion sickness using standardized subjective assessments and objective physiological endpoints; and strictly adhering to ethics approval and clinical trial registration requirements to lay the groundwork for subsequent evidence-based research. These early-phase trials should evaluate orexin-A not only as monotherapy but also in combination with established antiemetics (e.g., 5-HT_3_ or NK_1_ receptor antagonists) to explore potential synergistic benefits and define safety/tolerability profiles. Additionally, delivery system design should balance blood–brain barrier penetration with the risk of central effects, and early-phase studies should closely monitor potential adverse events (e.g., alterations in arousal levels, metabolic disturbances).

### 4.5 Limitations and future directions

There are only five animal studies in this review, resulting in limited evidence quality and necessitating caution in clinical extrapolation. These studies show heterogeneity in modeling methods, dosing regimens, administration routes, and outcome measurements, while insufficient reporting of randomization, blinding, baseline characteristics, and allocation concealment may introduce bias. The meta-analyses cover only a small number of studies and endpoints, and the gastric motility index shows moderate-to-high heterogeneity, warranting cautious interpretation. Restriction to English publications may also introduce language bias. Exclusion of unpublished data, such as conference abstracts and clinical trial registries, might lead to publication bias to publication bias. The following areas warrant further improvement:

Methodological Rigor: Adopt standardized CINV animal models, unify orexin-A administration protocols, and select consistent, validated endpoints (e.g., behavioral or physiological measures). Rigorously implement and transparently report randomization procedures, blinding, allocation concealment, and sample size calculations to improve comparability and reproducibility across studies.

Clinical Validation: Conduct rigorously planned early-phase clinical trials to assess the efficacy and safety of orexin-A in humans with motion sickness.

Mechanistic Elucidation: Investigate the molecular and cellular interactions between orexin-A and other neurotransmitters or signaling pathways to establish a foundation for more targeted therapeutic interventions.

Optimization of Administration Strategies: Explore more effective administration routes and dosing regimens, with particular attention to long-term safety and tolerability.

Model Diversification: Employ a wider range of animal models and pathological conditions to improve the generalizability and translational capacity of the results.

## 5 Conclusion

This review systematically compiled and examined existing animal model studies, providing preliminary evidence that orexin-A can alleviate motion sickness through coordinated action across multiple neural circuits. These findings offer novel insights into therapeutic development and establish a foundation for further investigations. Hower, because current evidence is limited to animal experiments, extensive future research is required to evaluate orexin-A’s safety, pharmacokinetic profile, and efficacy in humans, beginning with early-phase clinical trials. Advancing clinical research and further elucidating underlying mechanisms will be essential to translate orexin-A from bench to bedside.
